# A Case of Chorea: A Rare and Unusual Complication of Hyperglycemia

**DOI:** 10.7759/cureus.18730

**Published:** 2021-10-12

**Authors:** Nidhi Kaeley, Hari Prasad, Naveen Joseph, Anirban Ghosh Hazra

**Affiliations:** 1 Emergency Medicine, All India Institute of Medical Sciences, Rishikesh, IND

**Keywords:** involuntary movements, hyperglycemia, diabetes mellitus, chorea, blood sugar

## Abstract

We present a case of hemichorea in a patient with nonketotic hyperglycemia (NKH), a rare presentation of hyperglycemia. A 55-year-old female with diabetes presented to the emergency department with involuntary bilateral upper and lower limb movements for five days. The patient had a serum glucose level of 358 mg/dL (19.87 mmol/L) and improved after controlling blood sugar levels. When we encounter a case of chorea in the emergency department, high blood sugar levels are an essential underlying reversible etiology to be kept in mind.

## Introduction

India has a prevalence of diabetes of 8.7 [1]. Type 2 diabetes mellitus causes macrovascular and microvascular complications. The macrovascular complications include cardiovascular, cerebrovascular, and renal disorders [2,3]. Although neurologic complications are common in diabetes, chorea is a rare complication of nonketotic hyperglycemia (NKH) [4].

Chorea is a hyperkinetic movement disorder characterized by rapid nonpurposive movements of distal limbs and can involve the face and trunk. It can be caused by neurodegenerative, cerebrovascular, immunological, neoplastic, infectious, and metabolic diseases. Chorea following NKH has favorable outcomes after correction of hyperglycemia. Here, we present a case of an elderly female from a hilly region of Uttarakhand, manifesting as NKH chorea. Clinicians should be aware of this rare entity for early treatment.

## Case presentation

A 55-year-old female patient presented to the emergency department with complaints of acute onset generalized involuntary choreiform movements for the last five days. It started as bruxism and progressed to involve bilateral upper limbs and left lower limb and was associated with difficulty in speaking. It was noted that the movements resolved while the patient was sleeping. It was not associated with loss of consciousness, bladder or bowel incontinence, or weakness of any body part.

The patient had no history of fever, headache, vomiting, seizures, rash, drug intake, falls, and head injury and has been diagnosed with diabetes for 14 years on irregular treatment with insulin. There was no history of similar illnesses in the family. On examination, the patient was conscious, oriented, with a Glasgow Coma Scale (GCS) score of E4V5M6, blood pressure of 110/70 mmHg, respiratory rate of 20 cycles/min, SpO2 of 96% on room air, and pulse rate of 81 beats/min. The patient's bilateral pupils were reactive and of normal size and the patient had normal vesicular breath sounds. The abdomen was soft and nontender. Cardiovascular system examination revealed normal heart sounds. Regarding the central nervous system, choreiform movements in bilateral upper limbs and left lower limb were observed, and the power in all four limbs was 5/5. The tone was normal, and the bilateral plantar were flexor. Cranial nerve examination was normal. Cerebellar signs were absent. Video 1 (https://youtu.be/BPvT8tXDRC8) shows the involuntary movements of the patient.

**Video 1:** Nonketotic hyperglycemia (NKH) chorea. This video shows involuntary choreiform movements of the patient.

Investigations revealed mild anemia, and other blood tests were within normal limits (Table 1).

**Table 1:** Blood test results.

Parameters	Results	Reference range
Hemoglobin	9.6 g/dL (5.96 mmol/L)	12-15 g/dL (7.45-9.31 mmol/L)
Red blood cell count	3.74 × 10^6^/mcL (3.74 × 10^12^/L)	3.8-5.2 × 10^6^/mcL (3.8-5.2 × 10^12^/L)
White blood cell count	11.2 × 10^3^/mcL (11.2 × 10^9^/L)	4-11 × 10^3^/mcL (4-11 × 10^9^/L)
Platelets	216 × 10^3^/mcL (216 × 10^9^/L)	150-400 × 10^3^/mcL (150-400 × 10^9^/L)
Total bilirubin	0.29 mg/dL (4.96 µmol/L)	0.3-1.2 mg/dL (5.13-20.52 µmol/L)
Direct bilirubin	0.26 mg/dL (4.45 µmol/L)	0-0.2 mg/dL (0-3.42 µmol/L)
Alanine aminotransferase (ALT)	20.3 U/L (0.34 µkat/L)	0-35 U/L (0-0.58 µkat/L)
Aspartate aminotransferase (AST)	30 U/L (0.5 µkat/L)	0-35 U/L (0-0.58 µkat/L)
Alkaline phosphatase (ALP)	148 U/L (2.47 µkat/L)	30-120 U/L (0.5-2 µkat/L)
Serum albumin	3.47 g/dL (34.7 g/L)	3.5-5.2 g/dL (35-52 g/L)
Urea	40.1 mg/dL (6.67 mmol/L)	17-43 mg/dL (2.83-7.15 mmol/L)
Creatinine	1.05 mg/dL (92.84 µmol/L)	0.55-1.02 mg/dL (48.63-90.19 µmol/L)
Sodium	137.1 mEq/L (137.1 mmol/L)	136-146 mEq/L (136-146 mmol/L)
Potassium	5.04 mEq/L (5.04 mmol/L)	3.5-5.1 mEq/L (3.5-5.1 mmol/L)
Calcium	8.77 mg/dL (2.19 mmol/L)	8.8-10.6 mg/dL (2.2-2.64 mmol/L)

**Figure 1:**
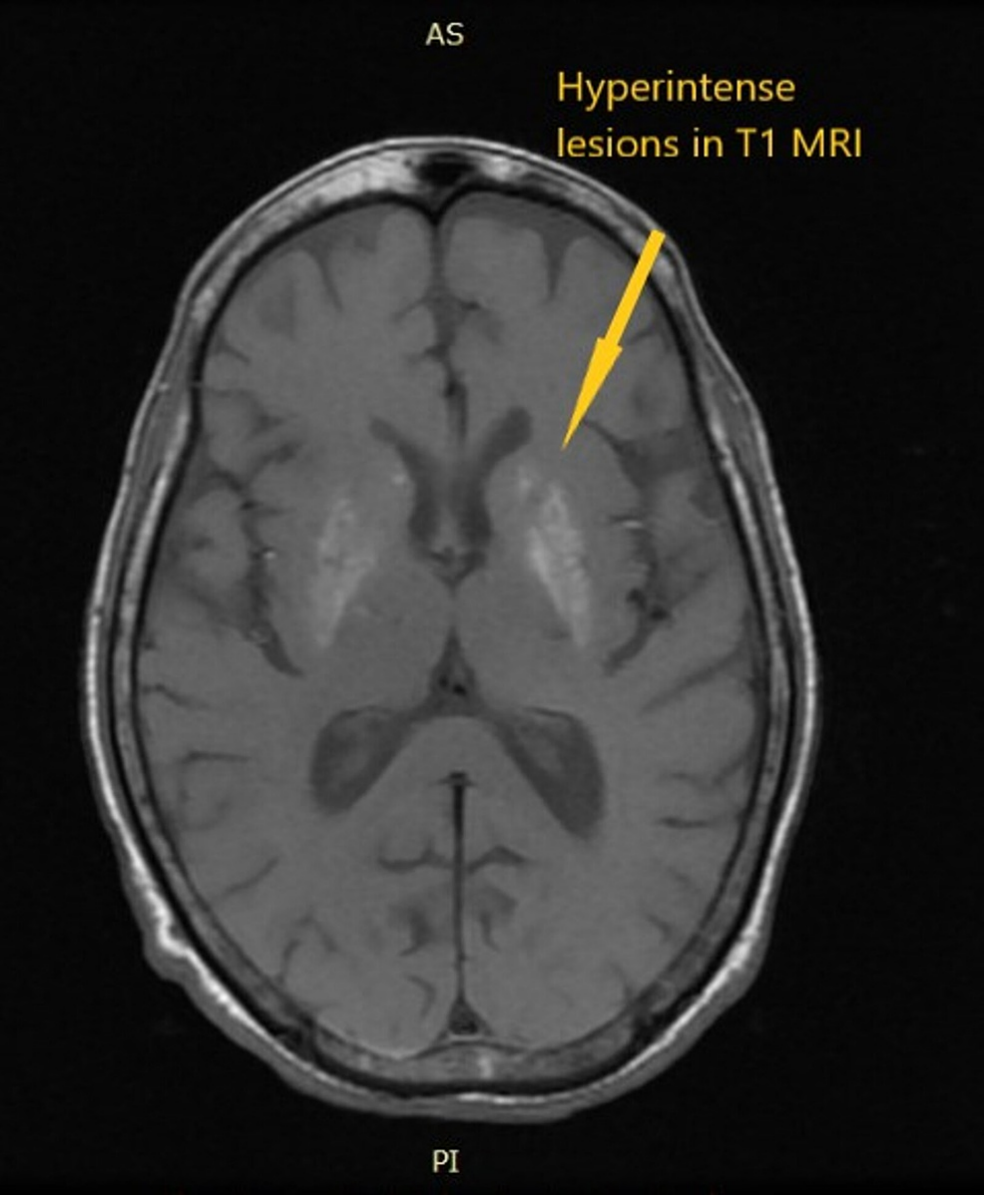
Hyperintense lesions in bilateral basal ganglia in T1 MRI.

Non-contrast computed tomography of the head revealed a tiny calcified granuloma in the right parietal lobe. Magnetic resonance imaging of the brain suggested a hyperintense lesion in the bilateral basal ganglia on T1, indicating a metabolic insult. Figure 1 shows a hyperintense lesion in bilateral basal ganglia on T1 MRI.

Random blood sugar level was 358 mg/dL (19.87 mmol/L) with negative urine ketones and normal arterial blood gas analysis (pH-7.354; pCO2-37.1mmHg; and HCO3-20.9 mmol/L). The patient's glycated hemoglobin (HbA1c) was 10.4%; thus, we made the diagnosis of NKH chorea. In the emergency department, isotonic saline (0.9% NaCl) at the rate of 10-20 ml/kg and insulin was administered to control blood sugar levels; and haloperidol (5 mg) was intravenously injected to control the involuntary movements of the patient. The patient was discharged with clonazepam (10 mg per tablet) along with insulin (subcutaneous). The patient is now asymptomatic with regular follow-up in the neurology outpatient department.

## Discussion

Chorea, athetosis, and ballism are the involuntary movements of the choreiform spectrum. NKH chorea encompasses a triad of chorea, hyperglycemia, and basal ganglia hyperintensity and is a rare complication of diabetes mellitus. The prevalence of NKH chorea is 1/100,000 and Bedwell, in 1960, has first reported NKH chorea as a rare clinical entity [2,3]. Oh et al. have reviewed 49 patients with NKH chorea from 1985 to 2001 where the study has revealed that the mean age of onset was 71.1 years, and the condition was more common in women than in men; moreover, they have concluded that MRI brain findings are reversible and improve with improvement in chorea [4].

The underlying pathogenesis of chorea in NKH chorea is attributable to hyperglycemia-induced basal ganglia dysfunction. Hyperglycemia leads to the stimulation of the anaerobic pathway in the brain, leading to Krebs cycle inhibition. The metabolic demand of the brain is fulfilled by converting gamma aminobutyric acid (GABA) to succinic acid, resulting in metabolic acidosis. In ketotic hyperglycemia, GABA is resynthesized; however, in NKH, both GABA and acetate are severely depleted, resulting in basal ganglia dysfunction [5]. Chorea is also observed in patients with hypoglycemia and rapid correction of hyperglycemia [6].

One radiological feature of NKH chorea is basal ganglia hyperintensity on T1-weighted MRI. A similar finding was observed in patients with hepatic encephalopathy, hypoglycemic coma, and post-cardiac arrest encephalopathy [7]. Most patients with nonketotic hyperglycemia respond well to the improvement of blood sugar levels; thus, strict blood sugar control is the mainstay of treatment. The other drugs that can treat chorea are dopamine antagonists such as risperidone, haloperidol, and GABA agonists such as clonazepam. NKH is a reversible cause of chorea, which should be timely identified and corrected. Moreover, hyperglycemia is a risk factor for cerebrovascular accidents and can lead to cerebrovascular insufficiency leading to striatum dysfunction, which manifests as chorea [8]. Table 2 shows the various case reports on NKH chorea.

**Table 2:** Various case reports on nonketotic hyperglycemic chorea. MRI, magnetic resonance imaging; NKH, nonketotic hyperglycemia.

Sl no	Study name	Year	Findings
1.	Chang et al. [9]	2010	Their study's clinical and imaging findings suggested that nonketotic hyperglycemia chorea may be due to reversible ischemia insult.
2.	Mahmoud et al. [10]	2014	First reported case of NKH chorea in a young white male with high T2-weighted (T2W) magnetic resonance signal in the basal ganglia. Chorea controlled with insulin and clonazepam.
3.	Ryan et al. [8]	2017	MRI putamen T1-hyperintensity is reportedly typical and it was only seen in 3/6 cases. Chorea controlled with dopamine blocking or depleting agents.
4.	Dong et al. [11]	2021	The study reported one uncommon case of NKH-chorea hemiballism and intracerebral hemorrhage that coincided in one patient.

## Conclusions

NKH chorea is a rare complication of uncontrolled diabetes mellitus and is more commonly seen in elderly females. It is one of the reversible metabolic causes of chorea; timely recognition and appropriate treatment of this condition will improve the patient's quality of life.
